# Exposure to the tsunami disaster, PTSD symptoms and increased substance use – an Internet based survey of male and female residents of Switzerland

**DOI:** 10.1186/1471-2458-8-92

**Published:** 2008-03-19

**Authors:** Stefan Vetter, Astrid Rossegger, Wulf Rossler, Jonathan I Bisson, Jerome Endrass

**Affiliations:** 1 Centre for Disaster and Military Psychiatry, University of Zurich, Zurich, Switzerland; 2 Psychiatric/Psychological Service, Criminal Justice System Canton of Zurich, Zurich, Switzerland; 3 Psychiatric University Hospital of Zurich, Research Unit for Clinical and Social Psychiatry, Zurich, Switzerland; 4 Department of Psychological Medicine, Cardiff University, Cardiff, UK

## Abstract

**Background:**

After the tsunami disaster in the Indian Ocean basin an Internet based self-screening test was made available in order to facilitate contact with mental health services. Although primarily designed for surviving Swiss tourists as well as relatives and acquaintances of the victims, the screening instrument was open to anyone who felt psychologically affected by this disaster. The aim of this study was to evaluate the influences between self-declared increased substance use in the aftermath of the tsunami disaster, trauma exposure and current PTSD symptoms.

**Methods:**

One section of the screening covered addiction related behavior. We analyzed the relationship between increased substance use, the level of PTSD symptoms and trauma exposure using multivariable logistic regression with substance use as the dependent variable. Included in the study were only subjects who reported being residents of Switzerland and the analyses were stratified by gender in order to control for possible socio-cultural or gender differences in the use of psychotropic substances.

**Results:**

In women PTSD symptoms and degree of exposure enlarged the odds of increased alcohol, pharmaceuticals and cannabis use significantly. In men the relationship was more specific: PTSD symptoms and degree of exposure only enlarged the odds of increased pharmaceutical consumption significantly. Increases in alcohol, cannabis and tobacco use were only significantly associated with the degree of PTSD symptoms.

**Conclusion:**

The tsunami was associated with increased substance use. This study not only replicates earlier findings but also suggests for a gender specificity of post-traumatic substance use increase.

## Background

Recent reports suggest that after natural disasters mental health problems and mental health needs pose a resource problem for primary care providers. About 4–5 percent of the survivors of a large-scale natural disaster can be expected to develop Posttraumatic Stress Disorder (PTSD) [1-5]. Among the survivors of Hurricane Katrina the rate of PTSD in survivors was even higher, with prevalence estimates of between almost 24% (severe pathology) and 38.6% (moderate pathology) [6].

However, large-scale disasters do not only affect primary victims but also individuals indirectly exposed to trauma [7] such as family members as well as friends of primary survivors, and also less severely affected community members [8-15].

Even though the prevalence of posttraumatic stress symptoms among an affected population decreases over time [16], there is evidence suggesting that disasters have other long-term mental health consequences such as major depression, anxiety disorders, functional disabilities, and disorders associated with substance abuse [17-20]. For the latter, there are several studies supporting this finding: After the 1995 bombing of the Murrah Federal Building a survey of residents of the Oklahoma City metropolitan area found increased alcohol use [21]. In the first 5 to 8 weeks after the September 11 terrorist attacks nearly 25% of the Manhattan residents acknowledged increased alcohol use, 9.7% increased cigarette smoking, and 3.2% increased marijuana consumption [22]. This finding was not limited to New York and the directly affected population. Stein et al. found increased use of alcohol and medication due to anxiety and worry about the terrorist attack in 38% of a nationally representative sample [14].

Posttraumatic stress disorder may be a risk factor for nicotine and drug use disorders [23]. It is, however, also important to understand that increased alcohol use after disasters does not automatically develop into substance abuse disorders [1,2,24-28]. Furthermore, there is also evidence that the prevalence of individuals consuming any substance does not increase, but that those persons who consumed substances before the traumatic event increase their consumption [13,25].

At present most of the research literature originates from North America. Unfortunately, there are no large-scale studies investigating increased substance use after natural disasters in Europe. The objective of this study was to evaluate the relationship between increased substance use and both exposure to the tsunami disaster as well as the level of self-reported PTSD symptoms with users of a web-based self-screening questionnaire in Switzerland. We hypothesized, that self reported PTSD symptoms were significantly associated with increased substance use.

## Methods

### Participants

The study sample consists of users of the "Online Self Evaluation Tool" (ONSET), an Internet based self-screening test, which was available after the Tsunami disaster in the Indian Ocean basin [29]. Between February 18th through August 31st, 2005 it was accessed 21,237 times, 4,161 persons registered on the site and 3,313 completed the entire self-screening for adults, of which 11.85% (n = 392) denied their consent for scientific analyses, resulting in a study sample of 2,921 adult screens. This study sample serves as the scientific basis for as series of research projects that investigate self reported PTSD symptoms of the participants. The 2,921 individuals originated from 61 different countries, although most were Swiss citizens (73%, n = 2,132).

All subjects, who were residents of Switzerland and were between 17 and 75 years old, were included in the study, resulting in a study sample of 2,269 subjects. The gender distribution was 900 (39.7%) female to 1369 (60.3%) male participants. The age median for women was 39.0 years (SD = 12.1) and for men 36.8 years (SD = 14.1).

### Data collection

Nearly 8 weeks after the Tsunami disaster ONSET was made available for survivors, relatives of victims and people who felt emotionally distressed by the media coverage and eyewitness reports of what happened [29]. It was designed to determine if people affected by the disaster had processed the events appropriately or if a consultation with a mental health professional for further assessment was indicated.

The online-test was designed from the start to be completely anonymous and at the end of the self-testing the user was asked whether he or she would consent to a scientific analysis of the data. Of the demographic variables only data concerning gender, age, civil status, education, citizenship, and country of residence was collected.

The level of exposure to the effects of the tsunami was assessed with 7 Yes/No questions: 1) witnessed the tsunami wave oneself, 2) hurt by the wave, 3) lost any family members (parents, siblings, children, partners etc.), 4) any family members (parents, siblings, children, partners etc.) were wounded and were subsequently hospitalized, 5) lost friends and acquaintances, 6) any friends and acquaintances were hurt and subsequently hospitalized, and 7) lost any possessions due to the tsunami.

The total number of positive answers was added up, resulting in a possible score of 0–7. To screen for possible PTSD pathology the Posttraumatic Stress Scale 10-item version (PTSS-10) with a Likert score between 0–3 [30] was used. The PTSS-10 was originally developed by the Division of Disaster Psychiatry, University of Oslo and the Norwegian Armed Forces Joint Medical Service in Oslo [31] as a clinical screening instrument to identify persons at risk of developing post-traumatic stress reactions.

Increased substance use was assessed in asking the participants, whether they had at all started or increased using a substance after the incident. Increased substance use was assessed with one question per substance (alcohol, tobacco, pharmaceuticals or cannabis/marihuana) with dichotomous answers (yes/no) asking if the users had increased their use.

### Statistical analyses

To assess the relationship between substance use, the level of PTSD symptoms and exposure to the tsunami, multivariable logistic regression analyses were carried out using the type of substance use as the dependent variable. In order to yield robust estimates, Bonferroni adjustment was used to control for multiple testing. The analyses were stratified by gender. All beta-weights of the logistic regression models were exponentiated to e in order to obtain odds ratios. Since one of the independent variable was continuous, the linearity in the logit was assessed using the fractional polynomial procedure in STATA SE 10.0. Goodness of fit was computed with the Hosmer-Lemeshow goodness of fit test. Outliers were investigated using a plot displaying leverage, Pearson residuals and influence statistics. All models were estimated with STATA SE 10.0.

## Results

### Exposure to traumatic events

Less than half of the users (n = 948, 41.78%) reported any direct contact with or any direct consequences from the tsunami disaster. 19.2% (n = 436) witnessed the tsunami wave themselves, 4.8% (n = 109) were injured, 4.7% (n = 106) lost a family member and 16.6% (n = 377) lost friends. 4.7% (n = 106) reported injured family members, 20.4% (n = 462) injured friends or acquaintances, and 10.6% (n = 241) lost property. The mean level of exposure was 0.81 (SD = 1.24) with a range from 0 to 7.

### Increased substance use after tsunami disaster

28.7% (n = 252) of the female and 23.1% (n = 398) of the male respondents reported increased substance use after the tsunami disaster. Women and men did not differ significantly in the prevalence of increased use, but there were gender specific differences in the chosen substance. Women reported an increased use of pharmaceuticals more frequently than men after the tsunami (women: 10.22% (n = 92); men: 6.57% (n = 90), chi^2 ^= 0.00). By contrast, men reported increased use of alcohol (women: 9.44% (n = 95); men: 14.83% (n = 203), chi^2 ^= 0.00) and cannabis (women: 2.11% (n = 19); men: 6.50% (n = 89), chi^2 ^= 0.00) more frequently. Table 1 provides an overview of the differences found between men and women with regards to increased substance use after the tsunami disaster.

**Table 1:** Increased substance use after tsunami disaster stratified for gender

	**All (N = 2269)**		**Women (N = 900)**		**Men (N = 1369)**		**Chi^2^**
**Increased use**	n	%	n	%	n	%	

**Any substance**	650	28.65	252	28.00	398	23.07	0.58
**Pharmaceuticals**	182	8.02	92	10.22	90	6.57	0.00
**Alcohol**	288	12.69	95	9.44	203	14.83	0.00
**Cannabis**	108	4.76	19	2.11	89	6.50	0.00
**Tobacco**	366	16.13	140	15.56	226	16.51	0.53

### Increased substance use, the level of PTSD symptoms and exposure to the tsunami disaster

In women increases used of alcohol was moderately correlated with increased use of pharmaceutical (r = 0.223) and increased use of tobacco (r = 0.207). In men, increased use of alcohol was considerably correlated with increased use of tobacco (r = 0.340) and cannabis (r = 0.240). The latter was furthermore correlated with increased use of pharmaceuticals (r = 0.346). These correlation scores of increased substance use after tsunami disaster stratified for gender are shown in table 2.

**Table 2:** Correlation scores of increased substance use after tsunami disaster stratified for gender

	**Alcohol**	**Tobacco**	**Pharmaceuticals**
**Women**			
**Tobacco**	0.207*		
**Pharmaceuticals**	0.223*	0.058	
**Cannabis**	0.138*	0.123*	0.053
**Men**			
**Tobacco**	0.340*		
**Pharmaceuticals**	0.155*	0.128*	
**Cannabis**	0.240*	0.346*	0.133*

Note:

*: p ≤ 0.05 (Bonferroni corrected)

Multivariable logistic regression showed that the severity of PTSD symptoms and the degree of exposure to the tsunami (represented by the sum score of "Yes" answers to the questions concerning exposure) were independently associated with any form of increased substance use as well as of increased use of pharmaceuticals.

In women both PTSD symptoms and degree of exposure enlarged the odds of increased alcohol use, increased consumption of pharmaceuticals and increased consumption of cannabis significantly. In the multivariable model an increased use of tobacco was only significantly associated with the degree of PTSD symptoms.

In men the relationship was more specific: PTSD symptoms – but not the degree of exposure – enlarged the odds of increased consumption of pharmaceuticals, alcohol, cannabis, and tobacco significantly.

In all the models, the PTSD symptoms proved to be linear in the logit. For the models that yielded a significant association between the independent and dependent variable. Hence, one change of unit in the former enlarges linearly the odds of the later. The results of the multivariable logistic regression analyses are shown in table 3.

**Table 3:** Increased substance use as a function of PTSD symptoms and exposure to incident: Multivariable regression analysis stratified for gender

		**Women**			**Men**		
**Increased use**		**OR**	**95% CI**		**OR**	**95% CI**	

**Any substance**	PTSD symptoms	*1.143	1.112	1.174	*1.108	1.088	1.129
	Exposure to incident	*1.215	1.080	1.367	1.106	1.000	1.223
**Pharmaceuticals**	PTSD symptoms	*1.172	1.125	1.221	*1.139	1.103	1.176
	Exposure to incident	*1.304	1.122	1.517	1.206	1.042	1.397
**Alcohol**	PTSD symptoms	*1.116	1.074	1.159	*1.110	1.086	1.135
	Exposure to incident	*1.177	1.010	1.372	1.008	0.892	1.139
**Cannabis**	PTSD symptoms	*1.148	1.057	1.246	*1.101	1.068	1.135
	Exposure to incident	*1.517	1.180	1.951	1.076	0.918	1.261
**Tobacco**	PTSD symptoms	*1.098	1.066	1.132	*1.078	1.055	1.100
	Exposure to incident	1.095	0.959	1.250	1.117	0.999	1.249

Note:

OR: Odds ratio.

95% CI: 95% confidence interval.

*: p ≤ 0.05 (Bonferroni corrected)

## Discussion

In this study, the prevalence of participants reporting increased substance use was 28.7%. Even though more men than women used ONSET, the female and male responders did not significantly differ in the prevalence of increased substance use. However, the results suggest that gender specific substance use exists. While women reported an increased use of pharmaceuticals, men reported increased use of alcohol and cannabis. Stratified multivariable regression analyses showed a multivariable association between any substance use and the severity of PTSD symptoms in both genders.

In women not only the PTSD symptoms but also the number of different exposures to the incident were significantly associated with increased alcohol use, increased consumption of pharmaceuticals, and increased consumption of cannabis, but not with increased tobacco consumption. In the male strata, the increased substances use was associated only with the severity of PTSD symptoms.

Overall, our study confirms the finding of increased substance use after traumatic experiences [14,17-22]. However, one limitation of the study is that we did not further explore the reasons for increased substance use. While some individuals might have used the substance as a self-medication, others might have consumed a substance as a coping mechanism. This would be the case if a person, who was emotionally distressed by the incident, sought relief in a behavior that has previously helped to reduce their level of distress. Even though our results do not permit us to answer this question directly, the finding from previous studies [13,25], that the number of people who consume specific substances does not increase, but that the amount consumed per person does, suggests, that the increased substance use is rather a coping mechanism than self-medication.

A second concern is that while increase of substance use was assessed it was not taken into consideration that persons might respond with a decrease in substance use. Thus no final inference is possible as to an overall increase of substance use after exposure to the Tsunami disaster.

A second limitation of the study was that we could not control for prior traumatogenic life events. Since ONSET was primarily designed as a public health screening instrument and not as a scientific research questionnaire, we tried to keep the instrument as short and user friendly as possible. Hence we did not explore pre-existing traumatic experiences or substance consumption habits at all. Furthermore, the data are cross-sectional and it is therefore impossible to determine whether the self reported mental health problems are the results of the Tsunami or represent the exacerbation of pre-existing problems.

A third concern is the composition of the study sample. Although the present study sample is of considerable size, the study population was a convenience sample and it was not possible to assess to what extent and with regard to which characteristics our sample was biased (when compared to the whole population of people affected by the tsunami). In order to use ONSET, the availability of the instrument had to be known, potential users needed Internet access, an email account, and basic computer skills. However, the results of our study are not meant to be interpreted as estimates of increased substance use in the general population, but rather serve as an estimate of online users in Switzerland.

A fourth limitation is, that we were not able to perform structured clinical interviews, but had to rely on self-reports. Also, the data were strictly anonymously assessed. Whether this increases or decreases the validity of the data in this context, cannot be answered. However, when analyzing the descriptive results, there were virtually no striking outliers such as unusually old age declarations that could have been interpreted as voluntary or involuntary false and misleading statements. Our results correspond to the findings of other studies in the field and we did not identify any response behavior that could be interpreted as a limitation of the internal validity of ONSET. We consider that there is a certain degree of empirical evidence that supports the overall validity of the study design and hence supports the use of an online assessment instrument for public mental health intervention strategies – especially when time matters.

## Conclusion

The major conclusion that results from our study is that we could replicate the finding that substance use increases after a large-scale disaster and that the type of increased substance use is gender specific. We therefore recommend further studies on the matter in order to corroborate our finding. Meanwhile we recommend to consider a routine assessment of increased substance use after large-scale incidents.

## Competing interests

The author(s) declare that they have no competing interests.

## Authors' contributions

SV developed ONSET and carried out the study design and was involved in writing the manuscript and has given final approval of the version to be published. AR performed the statistical analyses, was involved in writing the manuscript and helped to draft the manuscript. JE has given substantial contributions to conception, to analysis, interpretation of the data and writing of the manuscript. He has given final approval of the version to be published. WR and JB have been involved in revising the manuscript critically and have given final approval of the version to be published. All authors read and an approved the final manuscript.

## Pre-publication history

The pre-publication history for this paper can be accessed here:


